# Risk stratification improves outcomes in an osteoporosis fracture liaison service

**DOI:** 10.1007/s11657-026-01718-5

**Published:** 2026-06-12

**Authors:** Shejil Kumar, Lillias Nairn, Angela Bowman, Sally Dwyer, Ayanthi Wijewardene, Dahlia F. Davidoff, Matti L. Gild, Christian M. Girgis, Lyn March, Roderick J. Clifton-Bligh

**Affiliations:** 1 Department of Diabetes, Endocrinology & Metabolism, Royal North Shore Hospital, Sydney, NSW Australia; 2 Faculty of Medicine & Health, University of Sydney, Sydney, Australia NSW; 3 Cancer Genetics Laboratory, Kolling Institute, Royal North Shore Hospital, Sydney, NSW Australia; 4 Faculty of Medicine & Health, Discipline of Physiotherapy, University of Sydney, Sydney, NSW Australia; 5 Department of Physiotherapy, Royal North Shore Hospital, Sydney, NSW Australia; 6 Department of Rheumatology, Royal North Shore Hospital, Sydney, NSW Australia; 7 Sydney Musculoskeletal Health, Kolling Institute, Royal North Shore Hospital, Sydney, NSW Australia

**Keywords:** Fracture liaison services, Osteoporosis, Risk stratification

## Abstract

***Summary*:**

Selecting patients at higher baseline risk of fragility fracture may optimise clinical outcomes and cost-effectiveness of hospital fracture liaison services (FLS). In this cohort study, we found a risk-stratified approach led to a higher rate of treatment initiation and estimated number of fractures prevented compared to the traditional FLS strategy.

**Purpose:**

Hospital-based fracture liaison services (FLS) are cost-effective and reduce refracture risk. However, optimal FLS characteristics to maximise clinical and cost-effectiveness are uncertain.

**Methods:**

We reviewed data for FLS patients at Royal North Shore Hospital, Sydney (2015–2023). In 2018, the patient selection strategy was adjusted from a traditional approach (any fragility fracture, ≥ 50 years) to preferentially invite those either ≥ 60 years with any fragility fracture, or any presenting with hip and/or vertebral fractures. Cohorts entering the service pre-(FLS1) and post-this timepoint (FLS2) were compared regarding clinical characteristics, estimated fracture risk and pharmacotherapy initiation. Modelling was performed to estimate fractures averted.

**Results:**

The total cohort (*n* = 1903) was median 68-years-old and predominantly female (77%). Both cohorts were similar in sex distribution and prevalence of various fracture risk factors. The FLS2 cohort was older (median 69 vs 65 years, *p* < 0.001), more frequently presented with hip/vertebral fracture (21.4% vs 13.8%, *p* < 0.001), had higher Garvan-estimated 10-year fracture-risk (median 36.0% vs 27.4%, *p* < 0.001) and more frequently initiated pharmacotherapy (79.0% vs 64.6%, *p* < 0.001). In the overall cohort, strongest predictors of treatment initiation were older age, osteoporotic bone density, hip/vertebral fracture and female sex. Over 5 years, risk-stratified FLS was estimated to avert more osteoporotic (72 vs 44, *p* < 0.001) and hip fractures (20 vs 10, *p* < 0.001) per 1000 patients compared with traditional FLS.

**Conclusion:**

In this large hospital-based FLS study, a risk-stratified selection strategy was associated with more frequent pharmacotherapy initiation and estimated to avert more fractures; however, longitudinal assessment of treatment adherence and refracture rates is required to confirm utility.

**Supplementary Information:**

The online version contains supplementary material available at 10.1007/s11657-026-01718-5.

## Introduction

The prevalence of osteopenia, osteoporosis and fragility fractures is rising in our ageing population, with an already high global economic burden of fractures projected to increase [1–3]. In Australia, the total costs of osteoporosis/osteopenia are expected to double from 2023 to 2033 to greater than $8 billion, with acute hip fracture management the main source of expenditure. Despite availability of overall safe treatments effective at lowering fracture risk, low rates of appropriate pharmacotherapy initiation (~ 20%) after fragility fracture persist even in recent studies from well-resourced countries [4–6]. This is particularly alarming given the risk of refracture is disproportionately elevated within the first 1–2 years after an initial fracture [7, 8].

Fracture liaison services (FLS) are a cost-effective multidisciplinary strategy aimed to address this ‘treatment gap’ and considered a best practice approach worldwide [9, 10]. An intensive FLS systematically identifies patients with recent fragility fracture, assesses their risk of subsequent fracture and initiates pharmacotherapy where appropriate to lower refracture risk [11]. Meta-analyses have demonstrated efficacy of FLS in increasing bone density assessment, treatment initiation and persistence and reducing refracture risk compared with usual care in patients presenting with fracture [12–14].

However, there are concerns regarding resource and capacity constraints of hospital-based FLS, such that cost-effectiveness and added fracture-prevention benefit with expanding hospital-based FLS capacity has recently been challenged [15]. Fracture risk-reducing pharmacotherapy (e.g. bisphosphonates, denosumab, osteoanabolic agents) has been shown to reduce risk of vertebral fractures in postmenopausal women by approximately 30–70% and hip fractures and major osteoporotic fractures by approximately 30–50% compared with placebo in randomised controlled trials (RCTs) [16]. Hence, evidence-based osteoporosis pharmacotherapy treatment initiation is the key mechanism for an FLS to facilitate refracture prevention (in conjunction with falls prevention strategies). Most FLS models adopt a traditional approach of including all patients aged 50 years and older identified with recent fragility fracture [14]. However, optimal FLS operational characteristics for clinical and cost-effectiveness have not been established. We hypothesised a risk-stratified approach to patient selection would improve treatment initiation in a hospital-based FLS setting.

## Purpose

In this single-centre cohort study, a novel approach to selecting patients for FLS based on risk stratification by preferentially inviting patients 60 years or older or those with hip and/or vertebral fracture was compared to a historical FLS cohort using a traditional approach (age ≥ 50 years with any fragility fracture). Both cohorts were compared regarding clinical characteristics, estimated fracture risk and initiation of osteoporosis pharmacotherapy. Clinical predictors of treatment initiation were ascertained in the overall cohort. Modelling was performed to estimate the number of osteoporotic and hip fractures prevented over 5 years using the risk-stratified or traditional FLS approach.

## Methods

**Fig. 1:**
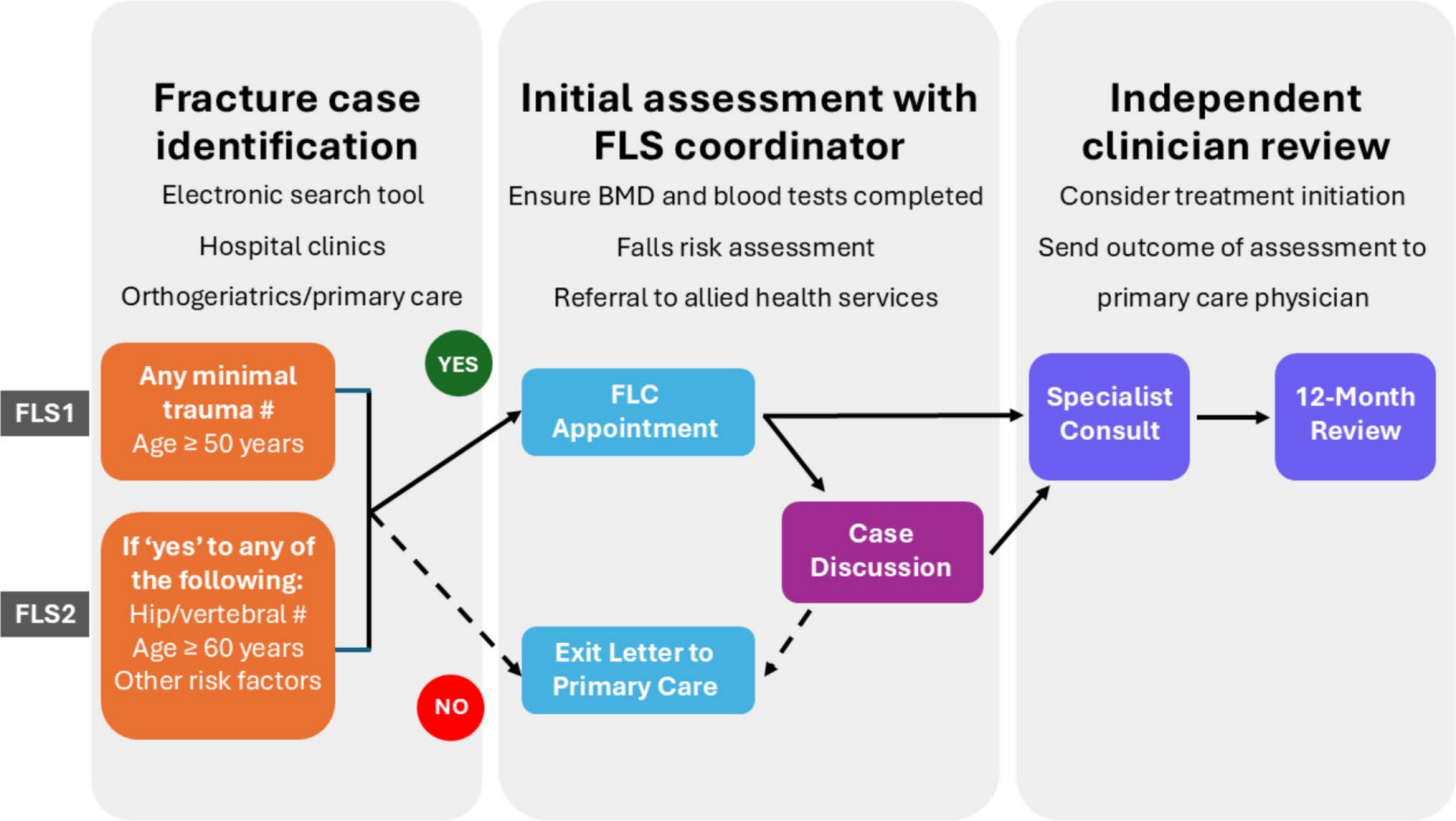
Fracture liaison service (FLS) model at Royal North Shore Hospital, Sydney

We conducted an observational cohort study of patients entering the FLS at Royal North Shore Hospital, Sydney, between November 2015 and June 2023. Cases of fragility fracture were detected by an automated electronic screening tool searching hospital electronic medical records and hospital radiology reports (allowing for detection of incidental vertebral fractures). Patients identified through the electronic screening tool were invited to the FLS with or without a specialist referral. Some patients entered the service via direct referrals from inpatient orthogeriatric service, hospital outpatient clinics (such as orthopaedic fracture, endocrinology and rheumatology clinics) and primary care. Between November 2015 and April 2018, FLS recruitment was based upon a traditional model whereby patients aged ≥ 50 years presenting with or detected to have any minimal trauma fracture were invited to the service. For this article, patients entering this traditional FLS model are referred to as the *FLS1 cohort* (Fig. 1). In May 2018, the patient selection strategy was recalibrated to optimise resource allocation. Subsequently, new patients with fragility fracture were only invited to the FLS if they met any of the following eligibility criteria: (i) age ≥ 60 years, or (ii) hip and/or vertebral fracture, or (iii) presence of risk factors for fragility fracture including parental hip fracture, current smoker, excess alcohol intake (> 3 standard drinks per day) and current prednisone use (> 5 mg/day or equivalent dose). Patients entering this risk-stratified FLS model are referred to as the *FLS2 cohort*. Patients were excluded from FLS if the index fracture was due to greater than minimal trauma, pathological/metastatic fracture, non-osteoporotic fracture (e.g. cervical spine, periprosthetic, fingers, toes, facial bones), atypical femoral fracture (referred directly to specialist clinic), already under osteoporosis specialist care, residential aged care facility resident, patient refusal, estimated life expectancy < 6 months or living outside the Northern Sydney Local Health District (NSLHD) catchment. For patients deemed ineligible, a letter would be sent to their primary care physician to provide recommendations for ongoing care.

FLS, fracture liaison service; FLC, fracture liaison service coordinator; #, fracture; BMD, bone mineral density. In the FLS1 cohort (November 2015–April 2018), any patient ≥ 50 years identified with minimal trauma fracture was invited to the FLS. After adopting a risk-stratified approach in May 2018 (FLS2 cohort), patients with minimal trauma fracture were only invited to the FLS if they met one of the following criteria: (i) ≥ 60 years, (ii) presenting with a hip and/or vertebral fracture or (iii) presence of risk factors for fragility fracture including parental hip fracture, prednisone use, excess alcohol intake and current smoker.

For eligible patients, a FLS coordinator conducted an initial review including education on osteoporosis and fragility fractures, ascertained falls risk, obtained dietary and physical activity history and ensured relevant investigations were completed (dual-energy X-ray absorptiometry bone mineral density (DXA BMD) scan; blood tests such as serum creatinine, calcium, phosphate and vitamin D concentration and workup for secondary causes of osteoporosis such as parathyroid hormone and thyroid stimulating hormone). The FLS coordinator also referred patients to allied health services or physical activity programmes as indicated. The FLS coordinator arranged a hospital outpatient consultation with a treating endocrinologist or rheumatologist attached to the service. Subsequent assessment and recommendation by the clinician were conducted according to routine clinical care and promptly communicated with the patient’s primary care physician in a consultation letter, after which routine 12-month review was arranged (or earlier depending on clinical circumstance). Only two out of ten clinicians attached to the FLS were aware of the shift from the traditional patient selection strategy to a risk-stratified approach. Clinical resources and personnel were similar between the different timeframes when using traditional vs risk-stratified FLS approach.

The FLS coordinator and treating clinicians entered data prospectively during or shortly after consultation into an electronic medical record form with data pooled directly into a study database. Only data collected from the initial assessment by the FLS coordinator and clinician are included in this study. Clinical datapoints included age (years) at entry into FLS, sex (male/female), height (cm), weight (kg), body mass index (kg/m^2^), index fracture site (hip/vertebral/non-hip non-vertebral), prior fragility fracture (yes/no), current osteoporosis pharmacotherapy (yes/no), frequency of falls in the past 12 months (0, 1–2 or ≥ 3) and various risk factors for fracture including parental hip fracture (yes/no), smoking status (current/past/never), excess alcohol intake ≥ 3 standard drinks/day (yes/no) and glucocorticoid use ≥ 5 mg/day prednisone or equivalent (current/past/never). DXA BMD values were recorded as both g/cm^2^ and corresponding T-scores (SD), the majority performed using Hologic Horizon® DXA system. Vitamin D was recorded as absolute value (nmol/L), and deficiency was defined as < 50 nmol/L. Estimated fracture risk assessment was performed using the Garvan risk calculator available at: https://fractureriskcalculator.com.au/calculator/, which incorporates sex, age, number of fractures since 50 years, number of falls in the past 12 months and femoral neck BMD values (either absolute g/cm^2^ or T-score (SD)). The calculator then provides an estimated 5- and 10-year risk of hip fracture (%) and any osteoporotic fracture (%). Fracture risk lowering pharmacotherapy initiation by treating clinicians was recorded, including oral bisphosphonates, intravenous zoledronic acid, subcutaneous denosumab, available osteoanabolic therapies (teriparatide, romosozumab) and menopausal hormone therapy.

Statistical analyses were performed using SPSS statistical software version 30.0. Patients already on osteoporosis pharmacotherapy at FLS entry were excluded from analyses. Comparisons were made between FLS1 and FLS2 cohorts for clinical and biochemical characteristics, DXA BMD values, Garvan-estimated fracture risk and treatment initiation. Categorical variables were expressed as *n* (%) and compared using chi-square test. Distribution of continuous variables was assessed using Shapiro–Wilk test for normality. Non-parametric variables were reported as median (IQR) and parametric variables as mean (± SD). Paired group comparisons for continuous variables were conducted using Wilcoxon sign-rank test and paired *t*-test, respectively. Between-group comparisons for continuous variables were conducted using Mann–Whitney *U* test and *t*-test, respectively. All analyses were two-sided, with *p*-value < 0.05 considered statistically significant. Binary logistic regression was performed to ascertain factors predicting treatment initiation in the overall cohort.

Modelling was performed to estimate fractures prevented per 1000 patients over 5 years when using either FLS1 or FLS2 strategy (see equation). Baseline fracture risk was taken as Garvan-estimated 5-year any osteoporotic and hip fracture risk (%). Relative fracture risk reduction with treatment initiation was taken as 40% for both osteoporotic and hip fractures based on meta-analysed RCT evidence [15]. To estimate fractures prevented, estimated fracture risk (%) for each patient was first divided by 100 to derive projected number of fractures per patient (*n*). When using the FLS strategy in either cohort, treatment effect (i.e. fracture risk reduction) was taken as 0.4 for patients initiating pharmacotherapy and 0 for those not initiating pharmacotherapy. Hence, median or mean fractures prevented were estimated in both FLS1 and FLS2 cohorts. Number needed to treat (NNT) to prevent one fracture was determined by the standard formula: NNT = 1/ARR; ARR = absolute risk reduction.

$$\mathrm{Fractures}\;\mathrm{prevented}\;\mathrm{over}\;5\;\mathrm{yrs}\;\left(\mathrm{per}\;1000\;\mathrm{patients}\right)\;=\overline\times\;\left(\frac{5\mathrm{yr}\;\mathrm{fracture}\;\mathrm{risk}\;(\%)}{100}\times\;\mathrm{treatment}\;\mathrm{effect}\right)\times1000$$

The study protocol was approved by the Northern Sydney Local Health District (NSLHD) Human Research Ethics Committee (HREC) (protocol number: 2019/ETH08156) including waiver of consent for analysis of prospective data collected on FLS patients during routine clinical care.

## Results

A total of 2141 patients attended the FLS between November 2015 and June 2023. Patients already on osteoporosis pharmacotherapy at FLS entry were excluded from data analyses (*n* = 238). The remaining cohort (*n* = 1903) was median 68 years old (IQR 60–76) and predominantly female (77.2%). Both the FLS1 (traditional model) and FLS2 (risk-stratified model) cohorts were similar in sex distribution and prevalence of various risk factors for fracture including parental hip fracture, smoking history, excess alcohol intake and vitamin D deficiency (Table 1). The FLS2 cohort was older, had more prevalent fracture history, more recent falls, was more likely to have osteoporotic BMD, more likely to present with hip/vertebral fracture and had higher Garvan-estimated 5- and 10-year risk of major osteoporotic and hip fracture. The FLS2 cohort was more likely to initiate pharmacotherapy (79.0% vs 64.6%, *p* < 0.001), most commonly zoledronic acid (26.1%), oral bisphosphonates (25.7%) and denosumab (25.0%), while osteoanabolic pharmacotherapy was uncommon.

**Table 1:** Cohort characteristics pre-(FLS1) and post-(FLS2) the amended selection criteria

Parameter	FLS1 (*n* = 603) *November 2015–April 2018*	FLS2 (*n* = 1300) *May 2018–June 2023*	*p*-value
Age, years at initial assessment (median (IQR))	65 (58–74)	69 (62–77)	< 0.001
Female, *n* (%)	479 (79.4%)	991 (76.2%)	0.121
BMI, kg/m^2^ (median (IQR))	25.3 (22.6–28.6) ^*n*=546^	25.1 (22.2–28.5) ^*n*=843^	0.253
Prednisone use (current), *n* (%)	9 (1.5%) ^*n*=591^	28 (2.2%) ^*n*=1272^	0.138
Excess alcohol intake (current), *n* (%)	55 (9.1%) ^*n*=591^	109 (8.6%) ^*n*=1266^	0.212
Cigarette smoking (current), *n* (%)	31 (5.2%) ^*n*=599^	77 (6.0%) ^*n*=1287^	0.706
Prior fragility fracture (pre-FLS), *n* (%)	112 (18.6%)	333 (25.6%)	< 0.001
Parental hip fracture, *n* (%)	78 (14.4%) ^*n*=541^	138 (14.1%) ^*n*=978^	0.870
Vitamin D deficiency, *n* (%)	88 (16.3%) ^*n*=540^	194 (17.4%)	0.581
Falls in last 12 months, *n* (%) - None - 1 or 2 - ≥ 3	393 (65.2%) 200 (33.2%) 10 (1.7%)	730 (56.2%) 530 (40.8%) 40 (3.1%)	< 0.001
Site of index fracture at FLS entry, *n* (%) - Vertebral - Hip - Non-hip, non-vertebral	66 (10.9%) 17 (2.8%) 520 (86.2%)	170 (13.1%) 108 (8.3%) 1,022 (78.6%)	< 0.001
DXA BMD T-score, SD (median (IQR)) - Lumbar spine - Total hip - Femoral neck	−1.3 (−0.3, −2.2) ^*n*=591^ −1.1 (−0.4, −1.7) ^*n*=406^ −1.6 (−1.0, −2.1) ^*n*=588^	−1.4 (−0.4, −2.3) ^*n*=1229^ −1.3 (−0.7, −2.0) ^*n*=1226^ −1.8 (−1.1, −2.4) ^*n*=1226^	0.421 < 0.001 < 0.001
DXA BMD status, *n* (%) - Normal-range - Osteopenia - Osteoporosis	116 (19.4%) 333 (55.7%) 149 (24.9%)	163 (13.0%) 671 (53.5%) 420 (33.5%)	< 0.001
Garvan 5-year fracture risk, % (median (IQR)) - Any major osteoporotic fracture - Hip fracture	14.0 (8.6–21.1) ^*n*=536^ 3.6 (1.6–7.4) ^*n*=528^	19.0 (11.4–31.0) ^*n*=867^ 6.0 (2.0–13.9) ^*n*=866^	< 0.001 < 0.001
Garvan 10-year fracture risk, % (median (IQR)) - Any major osteoporotic fracture - Hip fracture	27.4 (17.0–39.3) ^*n*=542^ 7.1 (3.1–14.6) ^*n*=528^	36.0 (23.0–54.0) ^*n*=879^ 11.0 (4.5–26.0) ^*n*=867^	< 0.001 < 0.001
Treatment initiation, *n* (%) - Any pharmacotherapy - Oral bisphosphonates - Zoledronic acid - Denosumab - Teriparatide/romosozumab - Menopausal hormone therapy - Nil pharmacotherapy	360 (64.6%) 79 (14.2%) 104 (18.7%) 170 (30.5%) 7 (1.3%) 0 (0%) 197 (35.4%)	918 (78.9%) 299 (25.7%) 304 (26.1%) 290 (24.9%) 23 (2.0%) 2 (0.2%) 245 (21.1%)	< 0.001

*FLS *fracture liaison service, *IQR* interquartile range, *BMI* body mass index, *DXA* dual-energy X-ray absorptiometry, *BMD *bone mineral density, *SD* standard deviation

Prednisone use defined as current exposure to 5 mg/day or more (or equivalent glucocorticoid dose). Excess alcohol defined as ≥ 3 standard drinks daily. Vitamin D deficiency defined as concentration < 50 nmol/L. BMD status defined based on lowest T-score at total lumbar spine, femoral neck and total hip. Left total hip and femoral neck BMD values were analysed (or right, if left unavailable). The total cohort (*n* = 1903) was obtained after excluding those already on osteoporosis pharmacotherapy at initial FLS consultation

*p* < 0.05 considered statistically significant

To assess secular trends in treatment initiation patterns, the same risk-stratified selection criteria (age ≥ 60 years or index hip and/or vertebral fracture) were applied to the FLS1 cohort which was then compared with baseline characteristics and treatment initiation rates in the FLS2 cohort. The adjusted FLS1 cohort had similar age, sex distribution, BMD T-scores, index fracture site, estimated fracture risk and treatment initiation rates (75.9% vs 78.9%, *p* = 0.219) compared with the FLS2 cohort (Supplementary Table 1).

In the overall cohort, several factors were univariately associated with initiating pharmacotherapy, including being in the FLS2 cohort, older age, female sex, lower BMI, current prednisone use, current cigarette smoking, prior fragility fracture (pre-FLS index fracture), being vitamin D-replete, falls in the last 12 months, vertebral/hip FLS index fracture, osteoporotic BMD and higher estimated fracture risk (Table 2).

**Table 2:** Patient characteristics based on pharmacotherapy treatment initiation

Parameter	Pharmacotherapy initiated (*n* = 1278)	Pharmacotherapy not initiated (*n* = 442)	*p*-value
FLS2 cohort	918 (71.8%)	245 (55.4%)	< 0.001
Age, years at initial assessment (median (IQR))	70 (63–78)	61 (55–69)	< 0.001
Female, *n* (%)	1016 (79.5%)	306 (69.2%)	< 0.001
BMI, kg/m^2^ (median (IQR))	24.6 (21.9–27.9) ^*n*=907^	26.7 (23.6–30.2) ^*n*=353^	< 0.001
Prednisone use (current), *n* (%)	33 (2.6%) ^*n*=1250^	2 (0.5%) ^*n*=433^	0.014
Excess alcohol intake (current), *n* (%)	102 (8.1%) ^n=1254^	46 (10.8%) ^n=427^	0.164
Cigarette smoking (current), *n* (%)	63 (5.0%) ^*n*=1266^	35 (8.0%) ^*n*=439^	0.004
Prior fragility fracture pre-FLS, *n* (%)	340 (26.6%)	67 (15.2%)	< 0.001
Parental hip fracture, *n* (%)	155 (15.4%) ^*n*=1004^	48 (12.8%) ^*n*=376^	0.212
Vitamin D deficiency, *n* (%)	176 (15.5%) ^*n*=1137^	82 (22.0%) ^*n*=372^	0.004
Falls in last 12 months, *n* (%) - None - 1 or 2 - ≥ 3	682 (53.4%) 566 (44.3%) 30 (2.3%)	338 (76.5%) 90 (20.4%) 14 (3.2%)	< 0.001
Site of FLS index fracture, *n* (%) - Vertebral - Hip - Non-hip, non-vertebral	187 (14.6%) 112 (8.8%) 979 (76.6%)	32 (7.2%) 2 (0.5%) 408 (92.3%)	< 0.001
DXA BMD T-score, SD (median (IQR)) - Lumbar spine - Total hip - Femoral neck	−1.7 (−0.7, −2.5) ^*n*=1214^ −1.5 (−0.9, −2.1) ^*n*=1045^ −1.9 (−1.4, −2.5) ^*n*=1208^	−0.5 (+ 0.5, −1.2) ^*n*=430^ −0.4 (+ 0.2, −0.9) ^*n*=351^ −0.8 (−0.3, −1.4) ^*n*=427^	< 0.001 < 0.001 < 0.001
DXA BMD status, *n* (%) - Normal-range - Osteopenia - Osteoporosis	72 (5.8%) 667 (54.0%) 497 (40.2%)	194 (44.5%) 225 (51.6%) 17 (3.9%)	< 0.001
Garvan 5-year fracture risk, % (median (IQR)) - Any major osteoporotic fracture - Hip fracture	20.0 (13.1–32.0) ^*n*=920^ 7.0 (3.3–15.0) ^*n*=914^	8.3 (5.2–13.0) ^*n*=346^ 1.0 (0.6–2.0) ^*n*=343^	< 0.001 < 0.001
Garvan 10-year fracture risk, % (median (IQR)) - Any major osteoporotic fracture - Hip fracture	37.5 (26.0–55.0) ^*n*=933^ 13.3 (6.7–28.0) ^*n*=915^	17.0 (11.0–25.5) ^*n*=350^ 2.3 (1.0–5.0) ^*n*=343^	< 0.001 < 0.001

*FLS* fracture liaison service, *IQR* interquartile range, *BMI* body mass index, *DXA* dual-energy X-ray absorptiometry, *BMD* bone mineral density, *SD* standard deviation

Prednisone use defined as current exposure to 5 mg/day or more (or equivalent glucocorticoid dose). Excess alcohol defined as ≥ 3 standard drinks daily. Vitamin D deficiency defined as concentration < 50 nmol/L. BMD status defined based on lowest T-score at total lumbar spine, femoral neck and total hip. Left total hip and femoral neck BMD values were analysed (or right, if left unavailable)

*p* < 0.05 considered statistically significant

Binary logistic regression was performed in the overall cohort to ascertain variables predicting pharmacotherapy initiation (Table 3). Significant predictors included increased age (OR 1.097), female sex (OR 2.810), lower BMI (OR 0.961), vertebral/hip index fracture site (OR 3.089) and osteoporotic BMD (OR 24.711).

**Table 3:** Binary logistic regression model assessing predictors of pharmacotherapy initiation

Independent variable	*p*-value	OR (95% CI)	
Age (↑)	< 0.001	1.10	(1.08–1.12)
Female sex	< 0.001	2.81	(1.86–4.24)
BMI (↓)	0.014	0.96	(0.93–0.99)
Current smoker	0.670	0.85	(0.41–1.77)
Prior fragility fracture (pre-FLS)	0.100	1.44	(0.93–2.21)
Vitamin D deficiency	0.063	0.66	(0.43–1.02)
Falls in last 12 months	0.085	1.44	(0.95–2.18)
Vertebral/hip fracture (FLS index fracture)	< 0.001	3.09	(1.72–5.54)
Osteoporotic BMD	< 0.001	24.71	(11.84–51.59)
Test	***p*-value**	***Χ***^**2**^	
Overall model likelihood ratio test (omnibus test)	< 0.001	367.16	

*CI* confidence interval, *FLS *fracture liaison service, *BMI* body mass index

Choice of independent variables for regression model based on variables with *p* < 0.05 for univariate analysis (see Table 2). Age (years) and BMI (kg/m^2^) were taken as continuous variables. The following variables were taken as dichotomous: cigarette smoking (current vs previous/never), prior fragility fracture (yes/no), vitamin D deficiency (yes/no), falls in the last 12 months (yes/no), index fracture site (hip/vertebral vs non-hip non-vertebral) and BMD status (osteoporotic vs normal/osteopenic). Garvan-estimated fracture risk was not incorporated in the model given dependence on other variables in the model (e.g. age, sex, number of fractures, number of falls, BMD). Similarly, FLS cohort status was not incorporated in the model. Prednisone use (current vs prior/never) was also removed from the model due to low numbers with current prednisone use. Binary logistic regression performed using SPSS version 30.0

The risk-stratified FLS approach (FLS2) was estimated to prevent a greater number of osteoporotic fractures (72 vs 44, *p* < 0.001) and hip fractures (20 vs 10, *p* < 0.001) per 1000 patients over 5 years compared with the traditional approach (FLS1) (Table 4). The FLS2 approach required a lower number needed to treat (NNT) to prevent one osteoporotic fracture (13 vs 22) and one hip fracture (50 vs 100).

**Table 4:** Projected 5-year fractures prevented using different FLS strategies

Treatment model	FLS1 (traditional)	FLS2 (risk-stratified)
*Any osteoporotic fractures*		
Fractures prevented per 1000 patients (median, IQR)	44 (0–80)	72 (24–124)**
Number needed to treat (NNT)	22	13
*Hip fractures*		
Fractures prevented per 1000 patients (median, IQR)	10 (0–27)	20 (2–56)**
Number needed to treat (NNT)	100	50

*FLS* fracture liaison service. Number needed to treat = 1/ARR. Absolute risk reduction (ARR) = number of fractures prevented/1000

**p-value < 0.001 using Mann–Whitney *U* test for independent comparison of non-parametric continuous data

## Discussion

In this large hospital-based FLS cohort study, we compared clinical and densitometric characteristics and treatment outcomes between two separate fracture cohorts who entered the FLS at different timepoints according to different patient selection criteria. We demonstrated that recalibrating patient selection from a traditional model (all patients aged ≥ 50 years with any fragility fracture) to a risk-stratified model (patients aged ≥ 60 years, or with hip and/or vertebral fractures) was associated with higher rates of treatment initiation. Since estimated fracture risk was also higher in the risk-stratified cohort, we estimated a significantly higher fracture risk reduction using a risk-stratified FLS compared with traditional FLS. Predictors of treatment initiation in the overall cohort included older age and hip/vertebral index fracture site (as well as female sex and osteoporotic BMD), consistent with the intent of our risk-stratified criteria. In the absence of longitudinal refracture assessment in our cohort, modelling of projected 5-year fracture rates predicted a risk-stratified FLS may result in a greater number of major osteoporotic and hip fractures prevented compared with a traditional FLS model of care. Further assessment in our cohort of treatment persistence, refracture rates and cost-effectiveness is required to confirm whether this risk-stratified FLS approach represents a more clinical and cost-effective strategy to meet demands of rising fracture prevalence while still operating within capacity constraints of a hospital-based FLS.

The overall benefits of FLS are well-described. FLS-based care is associated with more comprehensive skeletal assessment (e.g. DXA), more frequent post-fracture treatment initiation and, most importantly, reduction in refracture risk [12–14]. Our cohort of patients not on osteoporosis treatment at time of index fracture further highlights the existing osteoporosis treatment gap with 20–25% of patients having had prior fragility fractures. Meta-analyses (albeit of mostly non-randomised studies) have consistently shown a ~ 30% relative risk reduction for refracture with FLS vs non-FLS care, particularly in studies with longer follow-up of ≥ 2 years [12–14]. Several economic analyses globally (the UK, the USA, Australia, Asia) have demonstrated FLS strategies as cost-effective due to reduction in post-fracture care expenditure [17–20]. Although more intensive hospital-based FLS (such as that employed in our institution) are associated with greater fracture risk reduction and cost-effectiveness [21, 22], it is unclear whether resource constraints of such approaches can meet demands of rising fracture prevalence in our aging population. Indeed, the notion that the number of hospital-based FLS should be expanded was recently challenged [9, 15]. A system dynamics modelling study suggested only modest additional fracture prevention benefit and limited cost-effectiveness of increasing capacity and screening rates of hospital-based FLS programmes in Australia [15]. However, there were only minimal projected increases in number of patients assessed despite doubling and tripling the number of FLS programmes. Cost-effectiveness of expanding FLS was also underestimated as any cost-savings to the healthcare system by averting fractures were not considered. An alternative strategy would shift from specialist-led FLS to primary care to meet increasing demands of secondary fracture prevention. However, notwithstanding the importance of primary care in overall osteoporosis care and follow-up, there are concerns around treatment initiation and persistence if FLS strategies are only centred on primary care [4–6]. A pilot RCT suggested improved oral bisphosphonate adherence in primary care over 2 years providing treatment was initiated in an FLS setting [23]. A system dynamics modelling study further did not assess impact of changes in operational characteristics such as increasing treatment initiation rates [15], which might be achieved by risk-stratified selection criteria described herein to identify patients at higher refracture risk more likely to initiate pharmacotherapy.

To our knowledge, only one FLS study has assessed whether modified patient inclusion parameters could impact clinical efficiency. A retrospective Danish FLS study evaluated whether certain clinical risk factors (age, sex, bodyweight) could better predict patients who would be identified to have osteoporotic BMD using DXA [24]. A model selecting patients ≥ 50 years with bodyweight < 85 kg regardless of sex was the most accurate and would have been able to ‘exclude’ 20% of patients from having DXA assessment, while only missing osteoporotic BMD in 1% of patients. However, the purpose of an FLS is not to identify patients with an osteoporotic BMD. Although osteoporotic BMD was strongly associated with treatment initiation in our cohort, most fragility fractures at a population level (including in our cohort) occur in patients with osteopenic bone density [25]. Hence, excluding osteopenic patients from a hospital-based FLS strategy may not be suitable unless due to extreme resource limitations. Rather, the purpose of an FLS is to reduce refracture rates primarily by commencing appropriate pharmacotherapy with demonstrated RCT evidence for fracture risk reduction [16]. Therefore, a more suitable approach may be to identify clinical features associated with greater likelihood of initiating pharmacotherapy.

Our study demonstrated that modifying the FLS strategy to preferentially invite older patients or with hip/vertebral index fractures shifted the cohort to one with a significantly higher fracture risk and proportion of patients initiating treatment, and consequently, a predicted greater fracture prevention. Advanced age is a major risk factor for fragility fracture related to progressive microarchitectural degradation, bone density loss and increased risk of falls [26] and is incorporated into validated fracture risk calculators including FRAX and Garvan [27, 28]. A cross-sectional study of women presenting to an FLS in the UK found that in those with prior fracture history (*n* = 526), age ≥ 75 years was associated with twofold higher likelihood of being on treatment [29]. Meta-analyses of RCTs indicate similar fracture preventative effect of antiresorptives in postmenopausal women regardless of age [16, 30]; however, older patients are more likely to suffer hip fractures which carry the greatest post-fracture care expenditure [31]. Hence, it is plausible that targeting older patients for a hospital-based FLS may lead to greater treatment initiation rates and more cost-effective fracture risk reduction.

Preferentially selecting for patients with hip/vertebral fractures was a key component of our risk-stratified FLS approach, and index fractures at these sites were a strong predictor of treatment initiation. Hip/vertebral fractures (including radiographic vertebral fractures) carry the highest refracture risk, morbidity and fracture-related expenditure, hence representing a particularly at-risk group in need of prompt assessment and treatment initiation [31, 32]. Several studies have demonstrated greater treatment initiation rates in patients presenting with hip/vertebral rather than peripheral fractures [33–35]. In a UK-based FLS study, 85% of patients with hip fractures were on treatment at 6 months compared with 50% for proximal humerus fractures [33]. In a Canadian claims database, pharmacotherapy was 2.5-fold more likely to be commenced after VFs compared with forearm fractures [34]. Hip fractures (vs forearm fractures) were more associated with treatment initiation in a Spanish FLS study (89% vs 75%, *p* = 0.002) [35]. Most FLS target patients with any recent clinical fragility fracture and result in ~ 30% relative fracture risk reduction [12–14]; however, a recent meta-analysis demonstrates even greater fracture reduction (46%) in FLS programmes specifically for hip fracture patients [32]. Vertebral fractures are commonly under-represented in FLS cohorts due to reliance on their clinical presentation for detection [22]. In our FLS, patients were also identified by an electronic search tool reviewing hospital radiology reports, allowing for detection of radiographic vertebral fractures. This is crucial as incidentally detected vertebral fractures also carry increased risk of subsequent fracture and hence still represent a sentinel event [36], particularly given the thoracolumbar vertebrae are most sensitive to fracture risk reduction with osteoporosis pharmacotherapy [16]. Vertebral fractures are also associated with greater rates of subsequent hip fracture compared with other index fracture sites [37]. Therefore, there are several reasons why preferentially selecting patients with hip/vertebral fractures may improve clinical efficiency and resource allocation in a hospital-based FLS.

Although our study demonstrated a risk-stratified FLS strategy associated with more frequent initiation of osteoporosis pharmacotherapy, our study did not specifically assess treatment adherence or persistence. In real-world studies, adherence and persistence have been suboptimal with antiresorptives such as oral bisphosphonates (~ 50% persistence at 12 months) [38]. This is opposed to high rates of treatment persistence (~ 60–80%) in intensive FLS cohorts [22, 23], which may be even greater when dealing with hip/vertebral fracture patients and higher proportion of injectable therapies [39, 40]. Hence, although not specifically assessed in our study, we anticipated treatment persistence would have been high, given the characteristics of the FLS2 cohort. In the absence of longitudinal refracture data, we performed modelling to estimate fractures prevented over 5 years when using either a risk-stratified or traditional FLS approach. Using a traditional approach of including all patients ≥ 50 years with any fragility fracture, pharmacotherapy (initiated in 65%, median osteoporotic and hip fracture risk 14.0% and 3.6%) was projected to avert 44 osteoporotic fractures and 10 hip fractures per 1000 patients, However, using our risk-stratified approach, pharmacotherapy (initiated in 79%, median osteoporotic and hip fracture risk 19.0% and 6.0%) was projected to avert approximately twice as many fractures (72 osteoporotic fractures and 20 hip fractures). Greater cost savings with more fractures averted would be expected with the risk-stratified strategy; however, this was not explored in our study. Further insights into potential benefits of similar risk-stratifying approaches may be obtained if applied to large growing datasets of international FLS registries [41, 42].

Our fracture projection model has several limitations. We did not incorporate risk of multiple fractures per-patient or competing mortality risk (i.e. if patients died within 5-year follow-up prior to sustaining another fracture). Fractures prevented using either FLS approach are dependent on baseline fracture risk and treatment initiation rates and hence may have been overestimated given we utilised Garvan estimated fracture risk and assumed all patients initiating treatment would have persisted with and adhered to treatment. Certainly, estimated fracture prevention using our traditional approach (FLS1) was greater than that shown in other similar FLS programmes which have been modelled to prevent ~ 20 fractures [17, 43]. However importantly, our risk-stratified approach was estimated to avert almost twice as many fractures compared to a traditional approach. Treatment-specific fracture risk reduction efficacy was not incorporated into the model, due to inherent limitations in extrapolating this cohort to those studied in individual placebo-controlled trials. This study is further limited by its observational design leading to some missing data on important clinical characteristics. Given we utilised a historical FLS control group in this non-randomised study, we cannot exclude whether differences in patient characteristics, estimated fracture risk and treatment initiation were due to other unmeasured confounders or may reflect changing treatment practice in the institution over time, rather than specifically the recalibration of FLS patient selection. However, when applying our risk-stratifying selection criteria to the earlier cohort, similar rates of treatment initiation were observed suggesting higher treatment initiation rates in the later cohort were unlikely to be explained by secular trends in prescribing patterns.

## Conclusion

In this large cohort attending a hospital-based FLS, re-calibrating patient selection using a risk-stratified approach was associated with greater likelihood of pharmacotherapy initiation in subjects with higher estimated fracture risk. Modelling suggested this risk-stratified approach may lead to a greater number of fractures prevented. Further longitudinal assessment of treatment adherence, refracture rates and cost-effectiveness is required to confirm utility of this approach. Hospital-based FLS strategies targeting a more at-risk cohort whilst lower-risk patients are referred to primary care warrant further investigation and may improve clinical efficiency, resource allocation and cost-effectiveness of FLS to help meet the rising demands of secondary fracture prevention in our ageing population.

## Supplementary Information

Below is the link to the electronic supplementary material.

ESM 1 (DOCX 21.7 KB)

## Data Availability

The data underlying this study are not publicly available because sharing of individual patient-level data has not been approved by the ethics committee. Requests for access to un-identifiable data may be considered by the corresponding author on reasonable request and subject to approval by the ethics committee.
