# Supplementary material for: Risk stratification improves outcomes in an osteoporosis fracture liaison service
Source: Arch Osteoporos. 2026 Jun 12;21(1):90. doi: 10.1007/s11657-026-01718-5 (PMC13263213; doi:10.1007/s11657-026-01718-5)
Supplement: Supplementary file 1 — (DOCX 21.7 KB) [file 11657_2026_1718_MOESM1_ESM.docx]

**Supplementary Table 1 – Cohort characteristics when applying risk-stratifying criteria to the earlier cohort (FLS1-adjusted) compared with the prospectively risk-stratified cohort (FLS2)**

| **Parameter** | **FLS1 adjusted (n=416) *Nov 2015-April 2018*** | **FLS2 (n=1,300)**  ***May 2018-June 2023*** | **p-value** |
| --- | --- | --- | --- |
| Age, years at initial assessment (median (IQR)) | 70 (64 – 77) | 69 (62 – 77) | 0.052 |
| Female, n (%) | 325 (78.1%) | 991 (76.2%) | 0.426 |
| BMI, kg/m^2^ (median (IQR)) | 25.4 (22.7 – 28.6) ^n=371^ | 25.1 (22.2 – 28.5) ^n=843^ | 0.145 |
| Prednisone use (current), n (%) | 9 (2.2%) ^n=409^ | 28 (2.2%) ^n=1272^ | 0.436 |
| Excess alcohol intake (current), n (%) | 38 (9.3%) ^n=409^ | 109 (8.6%) ^n=1266^ | 0.548 |
| Cigarette smoking (current), n (%) | 15 (3.6%) ^n=414^ | 77 (6.0%) ^n=1287^ | 0.173 |
| Prior fragility fracture (pre-FLS), n (%) | 89 (21.4%) | 333 (25.6%) | 0.082 |
| Parental hip fracture, n (%) | 60 (16.4%) ^n=366^ | 138 (14.1%) ^n=978^ | 0.293 |
| Vitamin D deficiency, n (%) | 62 (16.5%) ^n=375^ | 194 (17.4%) | 0.706 |
| Falls in last 12-months, n (%) - None - 1 or 2 - ≥3 | 229 (55.0%)  179 (43.0%)  8 (1.9%) | 730 (56.2%)  530 (40.8%)  40 (3.1%) | 0.376 |
| Site of index fracture at FLS entry, n (%) - Vertebral - Hip - Non-hip, non-vertebral | 66 (15.9%)  17 (4.1%)  333 (80.0%) | 170 (13.1%)  108 (8.3%)  1,022 (78.6%) | 0.533 |
| DXA BMD T-score, SD (median (IQR)) - Lumbar spine  - Total hip  - Femoral neck | -1.3 (-0.2, -2.2) ^n=409^  -1.2 (-0.5, -1.8) ^n=283^  -1.7 (-1.2, -2.3) ^n=404^ | -1.4 (-0.4, -2.3) ^n=1229^  -1.3 (-0.7, -2.0) ^n=1226^  -1.8 (-1.1, -2.4) ^n=1226^ | 0.351 0.104 0.580 |
| DXA BMD Status, n (%)  - Normal-range - Osteopenia - Osteoporosis | 62 (15.0%) 238 (57.5%) 114 (27.5%) | 163 (13.0%)  671 (53.5%)  420 (33.5%) | 0.072 |
| Garvan 5-year fracture risk, % (median (IQR)) - Any major osteoporotic fracture - Hip fracture | 17.3 (12.4 – 25.0) ^n=368^  5.0 (2.7 – 9.3) ^n=364^ | 19.0 (11.4 – 31.0) ^n=867^  6.0 (2.0 – 13.9) ^n=866^ | 0.172 0.261 |
| Garvan 10-year fracture risk, % (median (IQR)) - Any major osteoporotic fracture - Hip fracture | 33.00 (24.4 – 45.1) ^n=371^  9.7 (5.0 – 18.0) ^n=364^ | 36.0 (23.0 – 54.0) ^n=879^  11.0 (4.5 – 26.0) ^n=867^ | 0.181 0.277 |
| Treatment initiation, n (%) - Any pharmacotherapy   - Oral bisphosphonates - Zoledronic acid - Denosumab - Teriparatide/Romosozumab - Menopausal hormone therapy   - Nil pharmacotherapy | 287 (75.9%)  52 (13.8%)  73 (19.3%)  155 (41.0%)  7 (1.9%)  0 (0%)  91 (24.1%) | 918 (78.9%)  299 (25.7%)  304 (26.1%)  290 (24.9%)  23 (2.0%)  2 (0.2%)  245 (21.1%) | 0.219 |

FLS = fracture liaison service; IQR = interquartile range; BMI = body mass index; DXA = dual-energy X-ray absorptiometry; BMD = bone mineral density; SD = standard deviation.
FLS1 adjusted cohort refers to those remaining in FLS1 cohort when retrospectively applying risk-stratifying selection criteria of age ≥60-years or index hip/vertebral fracture. Prednisone use defined as current exposure to 5mg/day or more (or equivalent glucocorticoid dose). Excess alcohol defined as ≥3 standard drinks daily. Vitamin D deficiency defined as concentration <50nmol/L. BMD status defined based on lowest T-score at total lumbar spine, femoral neck and total hip. Left total hip and femoral neck BMD values were analysed (or right, if left unavailable).
P<0.05 considered statistically significant.
